# Epigenome Editing for Cannabinoid Yield: Targets, Tools, and Priorities in *Cannabis sativa*

**DOI:** 10.3390/ijms27146299

**Published:** 2026-07-15

**Authors:** S. M. Ahsan, Sanjida Sultana Keya, Md. Injamum-Ul-Hoque, Nayan Chandra Howlader, Md. Rakib Hasan, Md Azizul Haque, Hossain Uddin Shekhar, Hyong Woo Choi, Md. Mezanur Rahman

**Affiliations:** 1 Department of Agriculture, Gopalganj Science and Technology University, Gopalganj 8100, Bangladesh; smvahsan@gmail.com (S.M.A.); injamumrassel@gmail.com (M.I.-U.-H.); nayanhowladar@gmail.com (N.C.H.); rakibhasanhlc@gmail.com (M.R.H.); 2 Institute of Cannabis Biotechnology, Andong National University, Andong 36729, Republic of Korea; 3 Department of Plant Medicals, Andong National University, Andong 36729, Republic of Korea; 4 Institute of Genomics for Crop Abiotic Stress Tolerance, Department of Plant and Soil Science, Texas Tech University, Lubbock, TX 79409, USA; skeya@ttu.edu; 5 Department of Biotechnology, Yeungnam University, Gyeongsan 38541, Republic of Korea; azizul@ynu.ac.kr; 6 Department of Biochemistry and Molecular Biology, Dhaka University, Dhaka 1000, Bangladesh; hossainshekhar@du.ac.bd; 7 Biosciences Division, Oak Ridge National Laboratory, 1 Bethel Valley, Oak Ridge, TN 37831, USA

**Keywords:** *Cannabis sativa*, cannabinoid biosynthesis, epigenome editing, histone modification, DNA methylation, glandular trichome

## Abstract

Cannabinoid content can vary several-fold across *Cannabis sativa* L. cultivars that carry functional, closely related *CBDA* synthase (*CBDAS*) and *THCA* synthase (*THCAS*) alleles, a difference that coding-sequence variation among functional alleles does not fully explain. Structural and copy-number variation at the synthase loci, the functional or pseudogenised state of synthase alleles, and linkage at the B locus account for much of the qualitative drug-versus-hemp chemotype; however, these genetic factors do not fully explain the quantitative, several-fold variation in cannabinoid content among cultivars that carry functional, near-identical synthase alleles. Three independent lines of evidence now indicate that chromatin-level regulation contributes to this variation. H3K4me3 and H3K56ac co-occupy the promoters and gene bodies of *THCAS*, *CBDAS*, *OLS*, and *OAC* exclusively in glandular trichome tissue while H3K27me3 marks the identical loci in vegetative tissues, indicating that a Polycomb-to-Trithorax chromatin switch is associated with trichome-specific cannabinoid gene expression, potentially independently of transcription factor availability. Progressive DNA hypomethylation accumulates during micropropagation in a cultivar-specific manner, with promoter-region differentially methylated positions reaching up to 22% by twenty subcultures, identifying DNA methylation maintenance as a candidate determinant of epigenetic stability in clonally propagated material, although the functional consequences for cannabinoid pathway gene expression and yield remain to be quantitatively established. In *Cannabis indica* cell suspension cultures, UV irradiation increases genome-wide DNA methylation (detected by MSAP, which does not resolve locus-specific changes) and elevates *CBDAS* transcript levels approximately 4-fold relative to non-irradiated controls. This coupling of an environmentally triggered methylation change to cannabinoid pathway gene expression in the *Cannabis* genus is suggestive, but the genome-wide MSAP signal and the use of *C. indica* cell cultures mean the locus-specific methylation change at the *CBDAS* promoter remains to be demonstrated. These findings identify specific, experimentally documented chromatin states as candidate targets for dCas9-effector intervention, including the H3K27me3 repressive state at cannabinoid loci that could be addressed by dCas9-KDM6A and the propagation-induced CG hypomethylation that could be addressed by dCas9-DNMT3A; these editing strategies remain hypotheses that have not yet been tested in *Cannabis*. We synthesize functional evidence from *Catharanthus roseus*, *Papaver somniferum*, and *Artemisia annua* demonstrating that equivalent chromatin switches control secondary metabolite yield in medicinal plants, evaluate which dCas9-effector architectures are most appropriate for each *Cannabis* chromatin target, and identify the critical mechanistic gaps that must be closed before epigenome editing can be rationally deployed for cannabinoid yield enhancement in *Cannabis*.

## 1. Introduction

*Cannabis* is among the fastest-growing pharmaceutical crops globally, with global legal *Cannabis* sales projected to reach USD 58 billion by 2028 [1]. Its therapeutic and commercial value is determined almost entirely by the content and composition of cannabinoids and terpenes accumulated in the secretory glandular trichomes of unfertilized female inflorescences [2,3]. Cannabidiol (CBD) and Δ9-tetrahydrocannabinol (Δ9-THC) are the two phytocannabinoids of greatest clinical relevance, with approved or late-stage therapeutic applications in epilepsy, neuropathic pain, anxiety disorders, and anti-inflammatory therapy [4,5]. Despite this pharmacological significance, cannabinoid content varies by as much as 10-fold across cultivars and more than 3-fold across developmental stages within the same variety [6]. No master transcriptional regulator of the cannabinoid pathway has been identified, no promoter architecture for its rate-limiting genes has been fully resolved, and no predictive model connects genotype to chemotype at the molecular level. A complementary explanation lies in epigenomics. The epigenome, defined as the complete set of DNA and histone modifications that regulate gene expression without altering the underlying DNA sequence, operates in plants through three interacting mechanisms: DNA methylation in the CG, CHG, and CHH contexts, covalent histone modifications such as H3K4me3, H3K27me3, and histone acetylation, and ATP-dependent chromatin remodeling, which together set which genes are accessible for transcription in a given cell type. This review asks whether these mechanisms govern cannabinoid biosynthetic gene expression and whether they can be harnessed to enhance cannabinoid yield. This question is especially pertinent in *Cannabis sativa*, where cannabinoid and terpene profiles vary several-fold across developmental stages and environments despite a stable genome, and even among cultivars carrying functional, near-identical synthase alleles, a plasticity that sequence-level variation alone does not explain. Whereas earlier *Cannabis* genomics centered on sequence-level features such as genome assembly, synthase gene structure, and copy-number variation, epigenome-scale studies remain scarce and have not functionally connected the chromatin state to cannabinoid pathway gene expression. This review consolidates that emerging evidence and evaluates how it can be advanced.

Cannabinoids are C_21_ terpenophenolic compounds, formed by the condensation of a resorcinol-type polyketide moiety with a monoterpene-derived isoprenoid unit, and they constitute a class of specialized metabolites largely restricted to *Cannabis* [7,8]. In the living plant, they accumulate predominantly as carboxylic (acidic) forms, such as cannabidiolic acid (CBDA) and Δ9-tetrahydrocannabinolic acid (THCA), which decarboxylate non-enzymatically to their neutral, pharmacologically active counterparts on heating, drying, or aging. More than one hundred and twenty distinct phytocannabinoids have been isolated from *C. sativa*, conventionally grouped into around eleven structural subclasses (the CBG, CBD, THC, CBC, CBND, CBE, CBL, CBN, CBT, cannabielsoin, and miscellaneous types), all of which derive from the single central precursor cannabigerolic acid (CBGA) and its propyl analog cannabigerovarinic acid through variation in side-chain length, cyclisation, and oxidation [7,8]. This shared biosynthetic origin coupled with extensive downstream tailoring accounts for the marked chemical diversity of the cannabinoid pool across tissues, developmental stages, and chemotypes. Beyond their pharmacological value to humans, cannabinoids appear to serve adaptive functions for the plant. They are synthesized and stored in the secretory cavity of capitate-stalked glandular trichomes that are most dense on the bracts of female inflorescences, a deployment consistent with protection of the developing seed. Acidic cannabinoids such as THCA and CBGA show antimicrobial and insecticidal activity in vitro, and a recent quantitative-genetic study reported that foliar cannabinoid concentration is inversely correlated with chewing-herbivore damage in the field and that *Trichoplusia ni* larvae grow more slowly and survive less well on high-cannabinoid leaves and on cannabinoid-amended diet, supporting a defensive role against herbivory [9]. Cannabinoid accumulation is also induced by ultraviolet-B exposure, suggesting an additional role in mitigating UV and oxidative stress, although the relative contributions of herbivore defense, antimicrobial protection, and abiotic-stress tolerance remain incompletely resolved [10]. Understanding how the plant regulates this defensive chemistry is therefore of both ecological and applied interest and motivates the focus of this review on the control of cannabinoid biosynthetic gene expression.

Cannabinoid biosynthesis converges on a single central precursor, cannabigerolic acid (CBGA), whose formation defines the metabolic commitment point for the entire pathway. CBGA is produced by condensation of olivetolic acid (OA) with geranyl pyrophosphate (GPP) by the prenyltransferase GOT at the surface of the secretory trichome [11]. OA itself is assembled from hexanoyl-CoA through a polyketide pathway requiring tetraketide synthase (TKS) and olivetolic acid cyclase (OAC) for correct aldol cyclisation; without OAC, TKS produces the shunt product olivetol rather than OA, demonstrating that OAC activity is the first rate-limiting checkpoint controlling carbon flux into the pathway [12]. GPP is supplied from the plastidic methylerythritol phosphate (MEP) pathway, and the intersection of two compartmentalized precursor streams at the trichome surface creates a metabolic bottleneck whose regulation is poorly understood [6]. CBGA is subsequently converted to cannabidiolic acid (CBDA) or Δ9-tetrahydrocannabinolic acid (THCA) by the substrate-competing enzymes CBDA synthase (CBDAS) and THCA synthase (THCAS), whose relative expression levels directly determine the CBD:THC chemotype ratio of a cultivar [13]. Both acids undergo spontaneous non-enzymatic decarboxylation to yield their pharmacologically active forms upon heating. The transcriptional output of four genes, *OAC*, *OLS*, *CBDAS*, and *THCAS*, controls cannabinoid identity and yield at every branch point of the pathway. What regulates the expression of these four genes at the chromatin, transcription factor, and post-translational levels remains the central unresolved question in *Cannabis* molecular biology.

Cannabinoid content varies by as much as 10-fold across cultivars, and at least part of this variation occurs among cultivars carrying functional, closely related *CBDAS* and *THCAS* alleles, a residual difference that coding-sequence variation among functional alleles does not fully explain [14]. Cannabinoid concentration increases more than five-fold between early and late floral stages, whereas transcript levels of core biosynthetic genes do not rise proportionally. This decoupling indicates that cannabinoid accumulation is shaped by regulation beyond transcript abundance, including post-transcriptional, translational, and enzyme-level control [3,6]. These observations require important genetic qualification, because several sequence-level factors beyond simple allelic identity are known to shape chemotype. The synthase genes reside within large, retrotransposon-rich, minimally recombining genomic regions that differ structurally between drug- and hemp-type alleles and contain one active enzyme alongside numerous pseudogenes [15]; copy-number variation at THCAS and CBDAS is common [16]; inactivating single-nucleotide polymorphisms, frameshifts, and pseudogenization determine whether a given synthase allele is functional and thereby set the qualitative drug-, hemp-, or CBG-dominant chemotype [17,18]; and the synthases are tightly linked to each other and to the aromatic prenyltransferase locus associated with total cannabinoid content [14,15]. What these structural, copy-number, and allelic-state determinants do not fully account for is the quantitative, several-fold variation in cannabinoid content among cultivars that share functional, near-identical synthase alleles, and it is this residual variation that the present review attributes in part to chromatin-level regulation. *CBDAS* and *THCAS* transcripts are essentially absent from leaves and stems but reach their highest levels in secretory glandular trichomes, implying that chromatin accessibility, not transcription factor availability, gatekeeps pathway expression in a cell-type-specific manner [19]. In *Catharanthus roseus*, jasmonate-responsive bHLH factors activate the alkaloid pathway promoters [20,21].

In *Papaver somniferum*, alkaloid biosynthesis is confined to specific cell types and cultivar-specific yield differences correlate with differential CHH methylation at biosynthetic loci [22,23,24], and in *Artemisia annua*, jasmonate-induced histone acetylation is implicated in activation of the artemisinin pathway [25,26]. In all three medicinal systems, a tissue-specific or environmentally responsive chromatin switch at rate-limiting pathway gene promoters controls metabolite yield independently of coding-sequence variation; *Cannabis* presents the same phenotypic profile, yet no equivalent chromatin-level analysis has been performed for any cannabinoid pathway gene.

In *Cannabis* glandular trichomes, the activating marks H3K4me3 and H3K56ac and the repressive mark H3K27me3 show reciprocal, tissue-specific distributions at cannabinoid and terpenoid loci [27]; micropropagation drives cultivar-specific accumulation of differentially methylated positions in promoter and intergenic regions [28]; and UV-B raises cannabinoid accumulation, although whether it acts through locus-specific methylation at pathway promoters is unresolved [10]. These lines of evidence, examined in detail in Section 3, leave open which enzymes write and erase these marks at pathway promoters and which signals recruit them to trichome chromatin.

Targeted epigenome editing with dCas9 fusion proteins has been demonstrated in plants with quantitative effects on pathway gene expression: dCas9-directed DNA demethylation activates ripening genes in tomato (*Solanum lycopersicum*) [29], dCas9-SunTag de novo methylation heritably silences target loci in *Arabidopsis* [30], and CRISPRa activates endogenous genes in *Nicotiana benthamiana* [31]. No equivalent experiment has been conducted in *Cannabis*: the chromatin states at OAC, OLS, CBDAS, and THCAS have not been functionally interrogated, and no dCas9-methyltransferase, dCas9-HAT, or CRISPRa approach has been applied to the cannabinoid pathway in any tissue [27].

**Figure 1:**
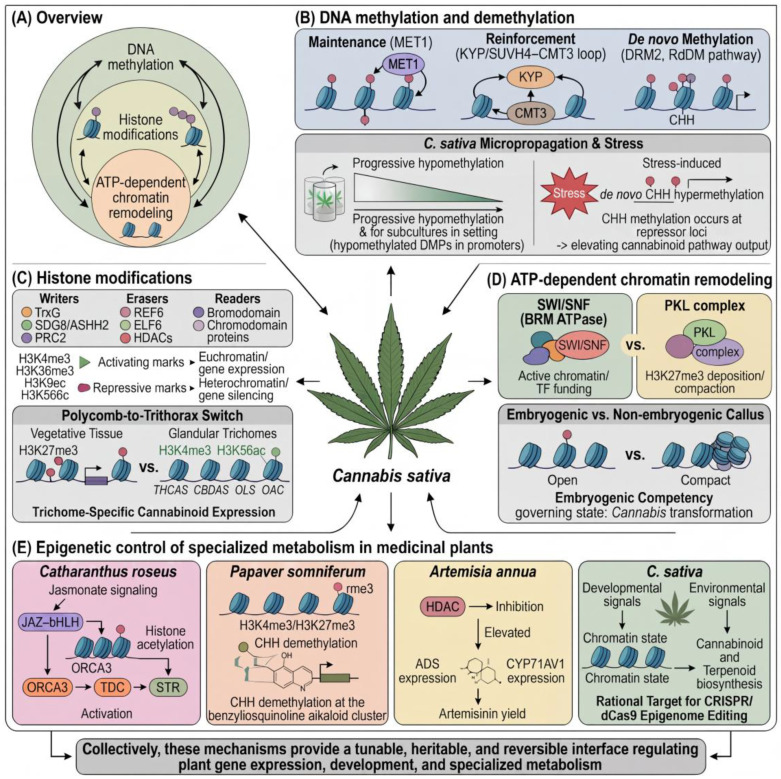
Three interconnected chromatin layers integrate developmental and environmental signals to regulate plant gene expression, development, and specialized metabolism. (**A**) Overview. Epigenetic regulation operates at the nucleosome and couples three interacting layers: DNA methylation, histone modifications, and ATP-dependent chromatin remodeling. (**B**) DNA methylation and demethylation. MET1 maintains CG methylation through replication, the KYP/SUVH4–CMT3 loop reinforces CHG methylation, and DRM2 establishes CHH methylation de novo through the RNA-directed DNA methylation (RdDM) pathway. During *Cannabis sativa* micropropagation, progressive hypomethylation accumulates across subcultures as heritable differentially methylated positions enriched in promoters, whereas stress induces de novo CHH hypermethylation at biosynthetic gene promoters to elevate cannabinoid pathway output. (**C**) Histone modifications. Writers (TrxG, SDG8/ASHH2, and PRC2), erasers (REF6, ELF6, and HDACs), and readers (bromodomain and chromodomain proteins) shape the modification landscape, which ranges from activating marks (H3K4me3, H3K36me3, H3K9ac, and H3K56ac) that establish euchromatin and gene expression to repressive marks (H3K27me3) that establish heterochromatin and gene silencing. In glandular trichomes, H3K4me3 and H3K56ac occupy *THCAS*, *CBDAS*, *OLS*, and *OAC*, whereas H3K27me3 marks the same loci in vegetative tissue, forming a Polycomb-to-Trithorax switch that gates trichome-specific cannabinoid expression. (**D**) ATP-dependent chromatin remodeling. SWI/SNF (with BRM as the catalytic ATPase) sustains open chromatin permissive for transcription factor binding, whereas the PKL complex promotes H3K27me3 deposition and chromatin compaction, without altering the underlying histone marks. The comparison of embryogenic and non-embryogenic callus illustrates how remodeling governs the open versus compact chromatin states that determine embryogenic competency relevant to *Cannabis* transformation. (**E**) Epigenetic control of specialized metabolism in medicinal plants. In *Catharanthus roseus*, jasmonate signaling acts through the JAZ–bHLH module and histone acetylation to activate ORCA3, TDC, and STR. In *Papaver somniferum*, the H3K4me3/H3K27me3 balance and CHH demethylation at the benzylisoquinoline alkaloid cluster set tissue-specific biosynthesis. In *Artemisia annua*, HDAC inhibition elevates ADS and CYP71AV1 expression and artemisinin yield. *C. sativa* follows the same paradigm, in which chromatin state integrates developmental and environmental signals to regulate cannabinoid and terpenoid biosynthesis, making it a rational target for CRISPR/dCas9 epigenome editing. Collectively, these mechanisms provide a tunable, heritable, and reversible interface regulating plant gene expression, development, and specialized metabolism.

This review therefore has a single aim: to assess whether chromatin-level regulation governs cannabinoid biosynthetic gene expression in glandular trichomes and whether it can be exploited to enhance cannabinoid yield. We map the histone modifications and DNA methylation patterns that demarcate tissue-specific cannabinoid gene expression, draw on functional evidence from model medicinal plants linking chromatin state to metabolite flux, evaluate which dCas9-effector architectures best fit each *Cannabis* chromatin target (Figure 1), and define the experimental priorities that must be met before epigenome editing can be deployed for cannabinoid crop improvement.

## 2. Chromatin Regulation of Plant Secondary Metabolism: Mechanistic Framework

The three regulatory layers, including DNA methylation, histone modification, and ATP-dependent chromatin remodeling, and their integration in medicinal plant secondary metabolism are summarized in Figure 1.

### 2.1. DNA Methylation Controls Transcriptional Access at Secondary Metabolite Gene Loci

Plant DNA methylation operates across three sequence contexts, CG, CHG, and CHH, each maintained by a biochemically distinct pathway and each with a different functional consequence for gene expression [32]. CG methylation is maintained through replication by MET1; its loss causes genome-wide CG hypomethylation and aberrant activation of numerous loci, consistent with MET1-dependent silencing operating broadly across the methylome [33]. CHG methylation is reinforced through a self-amplifying loop: the H3K9me2 histone mark deposited by KYP/SUVH4 recruits CMT3, whose methylation product in turn promotes further KYP/SUVH4 activity, creating a bistable chromatin state that locks gene loci in stable repression (Figure 1) [34]. CHH methylation is re-established de novo each cell cycle by DRM2 through the RNA-directed DNA methylation pathway, in which 24-nt siRNAs derived from Pol IV transcription of transposable elements guide sequence-specific methylation at flanking regions, suppressing readthrough transcription into adjacent genes [35]. Active demethylation is catalyzed by ROS1 and DME through base excision repair, replacing 5-methylcytosine with unmethylated cytosine at target loci [36]. In *Arabidopsis*, ROS1 preferentially targets TE loci proximal to protein-coding genes, preventing methylation spreading from TEs into flanking genic regions and maintaining their transcriptional competency, establishing that active demethylation is required for baseline expression of a substantial fraction of the gene-proximal transcriptome [37].

The functional relevance of DNA methylation for secondary metabolite biosynthesis has been documented in several crop species, though direct causal evidence linking specific methylation contexts to pathway tissue-specificity remains limited. In *Papaver somniferum*, comparative methylome analysis of high- and low-alkaloid cultivars reveals context-specific CHH hypomethylation in high-alkaloid stem tissues, with differentially methylated regions associated with cytochrome P450 enzymes, ABC transporters, and F-box proteins; however, the tissue-specific restriction of morphine pathway enzymes, including CYP80B1 to sieve elements and laticifers, is established through proteomics and in situ localization rather than through epigenetic manipulation [23,24]. In *Artemisia annua*, jasmonate-induced activation of *ADS*, *CYP71AV1*, and *DBR2* is mediated through a JAZ8–bHLH–ORA transcription factor cascade; whether CHG demethylation at pathway gene promoters co-regulates this response has not been experimentally tested [38]. In *Cannabis*, progressive global DNA hypomethylation accumulates during micropropagation in a cultivar-specific manner, with differentially methylated positions appearing preferentially in promoter and intergenic regions; the functional consequences for cannabinoid biosynthetic gene expression and yield remain to be quantitatively established [28]. Whether this hypomethylation reflects loss of DRM2-mediated CHH re-establishment, reduced CMT3–KYP feedback maintenance, or ROS1-driven active demethylation at pathway loci has not been determined.

### 2.2. Histone Modifications Set the Transcriptional State of Biosynthetic Gene Loci

H3K4me3, deposited by SET-domain TRITHORAX group methyltransferases, marks active transcription start sites and is required for RNA Pol II recruitment at target loci; *sdg2* loss-of-function in *Arabidopsis* causes genome-wide H3K4me3 depletion and broad misregulation of gene expression, confirming the essential role of this mark in maintaining transcriptional competency across the genome [39]. H3K27me3, deposited by Polycomb Repressive Complex 2 through its CLF, MEA, or SWN catalytic subunits, silences developmental genes during cell fate transitions and is removed by the H3K27 demethylase REF6 to permit transcriptional reactivation; ELF6 performs analogous demethylation at the *FLC* locus to prevent transgenerational inheritance of the vernalized state [40,41]. The opposing balance of H3K4me3 and H3K27me3 at a given locus constitutes a bivalent chromatin state whose resolution in one direction or the other determines whether the gene is expressed in a given tissue (Figure 1) [42]. H3K9me2, deposited by SUVH4/KYP, maintains constitutive silencing at transposable elements through a self-reinforcing loop with CMT3-mediated CHG methylation; disruption of either component simultaneously de-represses the other, confirming their mechanistic codependence [34]. In *Cannabis* glandular trichomes, H3K4me3 and H3K56ac co-occupy the promoters and gene bodies of cannabinoid biosynthetic genes, including *CBDAS*, *PT4*/*CBGAS*, and terpenoid synthases, specifically in floral trichome tissue, while H3K27me3 enriches at overlapping loci in vegetative tissues, establishing that the Polycomb–Trithorax balance at cannabinoid pathway gene promoters shifts predictably with the developmental transition to trichome-specific cannabinoid accumulation [27].

Histone acetylation at H3K9 and H3K14 opens chromatin at gene promoters by neutralizing the positive charge of lysine residues and reducing nucleosome–DNA affinity, creating a euchromatic state permissive for transcription factor binding [43]. GCN5/HAG1 is the primary H3K9/K14 acetyltransferase at light- and stress-responsive gene loci in plants, and its loss reduces inducible gene expression at target promoters in *Arabidopsis* [44]. HDA6 and HDA19 of the RPD3/HDA1 HDAC superfamily reverse these marks and restore repressive chromatin after stimulus withdrawal, providing temporal precision to transcriptional induction [45]. In *Cannabis sativa*, pharmacological HDAC inhibition with TSA, a genome-wide perturbation, is associated with altered expression of cannabinoid biosynthetic genes and altered accumulation of pathway precursors, and *CsHDA1*, *CsHDA5*, *CsHDA7*, and *CsSRT1* are co-expressed with cannabinoid pathway genes across tissues; these correlative data indicate that HDAC activity modulates the baseline output of the cannabinoid pathway, although locus-specific occupancy at pathway promoters has not been demonstrated [46]. Whether GCN5 or HDA6 orthologs directly occupy cannabinoid pathway gene promoters in *Cannabis* trichomes and whether their activities are modulated by developmental or environmental signals has not been investigated.

### 2.3. ATP-Dependent Chromatin Remodeling Controls Nucleosome Positioning at Pathway Gene Promoters

SWI/SNF and CHD family remodeling complexes use ATP hydrolysis to reposition or eject nucleosomes, exposing transcription factor binding sites without altering histone modification states, and acting upstream of transcriptional activation at developmental and secondary metabolite pathway loci (Figure 1) [47,48]. The SWI/SNF ATPase BRAHMA (BRM) maintains accessible chromatin at developmental gene promoters in *Arabidopsis*; genome-wide BRM binding analysis shows it directly binds and remodels nucleosomes at regulatory regions of stress- and development-responsive loci [49,50]. The CHD3 remodeler PICKLE associates with genes enriched for H3K27me3 and promotes H3K27me3 deposition at target loci, suppressing embryonic identity during germination through a mechanism in which chromatin remodeling and covalent histone modification act cooperatively [51]. In *Catharanthus roseus*, jasmonate treatment activates MIA pathway gene expression through JAZ–bHLH–ORCA cascades; whether SWI/SNF occupancy at pathway gene promoters precedes or follows transcription factor recruitment remains unresolved, as no chromatin remodeling occupancy data exist for MIA pathway loci [52]. No nucleosome positioning map, no SWI/SNF occupancy profile, and no CHD3 binding data exist for any cannabinoid pathway gene locus in *Cannabis*; recent ATAC-seq profiling confirms that chromatin accessibility at cannabinoid biosynthetic gene promoters correlates with expression differences between cultivars but does not identify the remodeling complexes responsible [53].

### 2.4. Epigenetic Chromatin Switches Control Secondary Metabolite Yield in Medicinal Plants

In *Catharanthus roseus*, jasmonate-induced activation of MIA biosynthetic genes *STR* and *TDC* is mediated through JAZ–CrMYC2–ORCA cascades; histone acetyltransferase activity contributes to transcriptional competency at pathway gene promoters, and HAT inhibition reduces pathway gene expression, as demonstrated for cannabinoid pathway orthologs in *Cannabis* [20,54]. In *Papaver somniferum*, tissue-specific restriction of alkaloid pathway enzymes, including STORR, CYP80B1, and T6ODM, to sieve elements and laticifers is established through cell-type-specific transcriptional control; comparative methylome analysis reveals cultivar-specific CHH hypomethylation in high-alkaloid tissues, though direct evidence linking DNA methylation at TE-flanking regions to histone modification state switching at pathway gene promoters has not been published [23,24]. In *Artemisia annua*, an *AaHAT* gene family of 11 members is expressed in glandular trichomes and contains MeJA-responsive *cis*-elements, suggesting that histone acetylation co-regulates jasmonate-induced pathway output; direct pharmacological evidence using HAT or HDAC inhibitors at *ADS* and *CYP71AV1* promoters remains to be published [26]. These three systems establish a shared mechanistic principle: rate-limiting pathway gene loci are subject to reversible chromatin state control by histone acetyltransferases, HDACs, and DNA methylation machineries, and their transcriptional activation requires coordinated chromatin remodeling. In *Cannabis*, cannabinoid biosynthetic genes are regulated with trichome-specific and developmentally gated transcriptional precision; HAT inhibition is associated with reduced cannabinoid pathway gene expression and product accumulation in hemp inflorescences, consistent with, though not yet demonstrating, locus-specific acetylation control at these promoters, and trichome-specific H3K4me3 and H3K56ac landscapes demarcate active cannabinoid gene loci (Figure 1) [27,54]. Whether the same molecular switches operate at *OAC*, *OLS*, *CBDAS*, and *THCAS* at the level of locus-specific histone modification dynamics in response to developmental and environmental signals is the central unresolved question this review addresses (Table 1).

## 3. Epigenetic Regulation of Cannabinoid Biosynthesis in *Cannabis sativa*: Current Evidence

**Figure 2:**
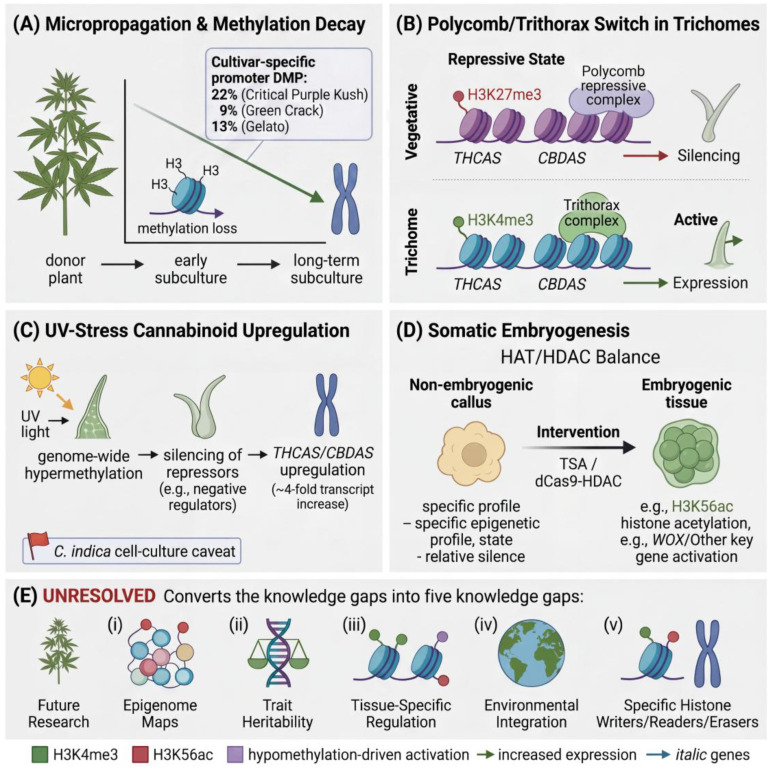
Current experimental evidence defines the empirical foundation and mechanistic gaps of *Cannabis sativa* epigenomics. (**A**) DNA methylation dynamics during micropropagation. Progressive DNA hypomethylation accumulates from the donor plant through early to long-term subculture in a cultivar-specific manner, representing a quantitative source of epigenomic instability whose effect on cannabinoid output remains untested. MET1 maintains CG methylation during vegetative propagation, and its reduced fidelity under in vitro conditions permits progressive epigenetic drift. Differentially methylated positions accumulate preferentially in promoter regions, reaching 22%, 9%, and 13% in the cultivars Critical Purple Kush, Green Crack, and Gelato, respectively. (**B**) Reciprocal Polycomb-to-Trithorax switch at cannabinoid biosynthesis loci. In vegetative tissue, the repressive mark H3K27me3 and the Polycomb repressive complex occupy the *THCAS* and *CBDAS* loci, maintaining silencing. In glandular trichomes, the activating mark H3K4me3 and the Trithorax complex occupy the same loci, driving expression, defining experimentally validated targets for CRISPR/dCas9 epigenome editing [27]. (**C**) UV-induced cannabinoid upregulation. In *Cannabis indica* cell suspension cultures, UV irradiation induces genome-wide hypermethylation, which is proposed to silence transcriptional repressors and thereby release *THCAS* and *CBDAS*, yielding an approximately fourfold transcript increase [55]. Because these data derive from *C. indica* cell cultures rather than *C. sativa* trichomes, locus-specific validation in the relevant tissue and species is required (flag). (**D**) Epigenetic control of somatic embryogenesis through the HAT/HDAC balance. Non-embryogenic callus displays a relatively silenced epigenetic profile, whereas intervention with trichostatin A (TSA) or dCas9-HDAC constructs shifts the balance toward embryogenic tissue, marked by increased histone acetylation (for example, H3K56ac) and activation of key genes such as *WOX*, potentially relieving the genotype-dependent regeneration barriers that constrain *Cannabis* biotechnology [46,56]. (**E**) Five unresolved knowledge gaps that constrain rational dCas9-effector design and define priorities for future research: (i) base-resolution epigenome maps, (ii) trait heritability, (iii) tissue-specific regulation, (iv) environmental integration, and (v) identification of specific histone writers, readers, and erasers. In all panels, green denotes H3K4me3, red denotes H3K56ac, and purple denotes hypomethylation-driven activation; green arrows denote increased expression and italics denote gene names.

Five studies now define the empirical foundation of *Cannabis* epigenomics, spanning DNA methylation dynamics during micropropagation, the histone modification landscape of glandular trichomes, environmental epigenetic responses, and chromatin dynamics during in vitro organogenesis. Together, they establish that epigenetic mechanisms operate at every level of *Cannabis* biology relevant to cannabinoid yield. They also expose five specific mechanistic gaps whose resolution is required before epigenome editing can be rationally designed for cannabinoid enhancement (Figure 2; Table 1).

### 3.1. Progressive DNA Hypomethylation Accumulates During Micropropagation

Genome-wide 3D-GBS and enzymatic methyl sequencing of three *Cannabis* cultivars, including Critical Purple Kush, Green Crack, and Gelato, subcultured across 20 subcultures over 60 weeks, revealed that differentially methylated positions accumulate with subculture number in a cultivar-specific pattern, with the majority of mutations arising during culture initiation and the first five subcultures [28]. The rate and genomic distribution of epimutation differed significantly among cultivars, promoter-region DMPs reaching 22% in CPK, 9% in GC, and 13% in GEL, demonstrating that CG and CHG methylation maintenance fidelity varies with genotype under identical tissue culture conditions (Figure 2) [28]. Progressive DNA hypomethylation has also been documented in *Cannabis* shoots during in vitro subculture using MSAP-Seq, confirming that epigenetic drift is a reproducible outcome of in vitro propagation; however, the direct functional consequences for cannabinoid biosynthetic gene expression and yield have not been quantitatively established [28]. Whether the observed hypomethylation reflects impaired DRM2-mediated CHH re-establishment, reduced CMT3–KYP feedback loop activity, or enhanced ROS1-driven active demethylation at pathway loci has not been determined.

### 3.2. Trichome-Specific Histone Modification Switches Activate Cannabinoid Pathway Gene Loci

ChIP-seq and RNA-seq analysis of glandular trichome, leaf, and stem tissues from *Cannabis sativa* mapped the active and repressive histone modification landscape at cannabinoid and terpenoid biosynthetic gene loci across three tissue types, providing the first genome-wide histone modification maps for *C. sativa* glandular trichomes [27]. H3K4me3 and H3K56ac co-occupy the promoters and gene bodies of cannabinoid biosynthetic genes, including *CBDAS*, *PT4*/*CBGAS*, and terpenoid synthases, specifically in glandular trichome tissue but are absent at the same loci in leaf and stem, establishing that trichome-specific transcriptional activation is marked by a permissive chromatin state absent in vegetative tissues (Figure 2) [27]. H3K27me3 and H2A.Z show the reciprocal pattern, co-localizing in the gene bodies of silenced cannabinoid pathway genes in vegetative tissues and depleted in trichomes, indicating that the developmental transition to trichome identity involves a coordinated Polycomb-to-Trithorax switch at pathway gene promoters (Figure 2) [27]. Trichome-specific intergenic regions of high H3K56ac enrichment without corresponding H3K4me3 at transcription start sites identify candidate putative enhancer elements whose role in co-expression of cannabinoid and terpenoid pathway gene clusters has not been functionally tested [27]. Genes encoding starch and sucrose metabolism enzymes, which supply precursor pools for cannabinoid biosynthesis, also show trichome-specific H3K4me3 and H3K56ac enrichment; complementary ATAC-seq profiling further shows that chromatin accessibility at fatty acid biosynthesis and trichome initiation gene promoters, rather than at core cannabinoid synthase promoters, is the primary epigenetic driver of cannabinoid yield differences between cultivars [27,53]. The identities of the transcription factors that read the H3K4me3 marks at cannabinoid pathway gene promoters, and the upstream signals that recruit responsible Trithorax methyltransferases to these loci during trichome differentiation, remain unknown.

### 3.3. UV Irradiation Induces Genome-Wide DNA Hypermethylation and Cannabinoid Gene Upregulation in Cannabis indica

Exposure of *Cannabis indica* cell suspension cultures to UV radiation at a 4 h photoperiod increased genome-wide DNA methylation, as detected by methylation-sensitive amplified polymorphism analysis, and elevated *CBDAS* and *THCAS* transcript levels approximately 4-fold relative to non-irradiated controls, with progressive upregulation of *OAC* and *OLS* also observed (Figure 2). This result, observed in *C. indica* rather than *C. sativa*, demonstrates that environment-triggered DNA methylation changes are mechanistically coupled to cannabinoid pathway gene expression in the *Cannabis* genus, though the species distinction warrants caution in extrapolation. The MSAP method used detects methylation at CCGG sites genome-wide but cannot resolve whether the hypermethylation occurs at cannabinoid gene promoters directly or at flanking repressor loci whose silencing releases pathway gene expression. In *Arabidopsis*, the DNA demethylase ROS1 preferentially targets transposable element loci proximal to protein-coding genes, preventing methylation spreading into flanking genic regions; whether analogous UV-B-responsive ROS1-mediated demethylation at pathway gene-adjacent TE loci occurs in *Cannabis* is unknown [37]. Whole-genome bisulfite sequencing at single-base resolution in *C. sativa* tissues subjected to UV-B, drought, and heat stress is required to determine whether stress-responsive methylation changes at cannabinoid pathway gene promoters constitute a conserved environmental induction mechanism.

### 3.4. Chromatin Remodeling Governs the Embryogenic Competency Transition During Cannabis Organogenesis

Transcriptomic comparison of non-embryogenic and embryogenic *Cannabis* callus identified 247 differentially expressed epigenetic regulator genes spanning DNA methyltransferases, histone-modifying enzymes, chromatin remodeling complex subunits, and small RNA pathway components, indicating that the acquisition of embryogenic competency in *Cannabis* involves wholesale reprogramming of the chromatin regulatory machinery (Figure 2) [57]. HAT and HDAC activities are central to this transition: chromatin remodeling factors, including CHROMATIN REMODELING 35 (CRF35), mediate crosstalk between H3K27me3 and HDAC activity to repress embryogenesis-related transcription factors (*LEC1*, *LEC2*), and their upregulation in embryogenic calli correlates with the repression of somatic embryogenesis progression [57]. This mechanism is consistent with findings across multiple plant species, where HDAC inhibition with trichostatin A de-represses H3 acetylation at embryogenic loci and significantly enhances somatic embryogenesis frequency, including in *Arabidopsis* and *Cannabis* root explants [46,58]. The practical implication is direct: pharmacological or dCas9-HDAC-based modulation of histone deacetylase activity at embryogenic competency loci may improve the genotype-dependent transformation and regeneration efficiencies that currently constrain *Cannabis* biotechnology.

### 3.5. Five Mechanistic Gaps Define the Frontier for Cannabis Epigenome Editing

Five mechanistic gaps define the frontier for *Cannabis* epigenome editing, and their closure is required before dCas9-effector strategies can be rationally designed for cannabinoid yield enhancement. First, no comprehensive, whole-genome base-resolution methylome exists for any cannabinoid-relevant *Cannabis* tissue. The available base-resolution methylation data (enzymatic methyl sequencing of micropropagated shoots; [28]) derive from a reduced-representation 3D-GBS protocol that samples only a fraction of the genome and were generated in whole shoot tissue rather than glandular trichomes. Whole-genome bisulfite sequencing or EM-seq covering CG, CHG, and CHH contexts in glandular trichomes, leaf epidermis, and vascular tissue has not been published, and the RdDM pathway’s role at transposable element-proximal cannabinoid pathway genes remains entirely unexplored [32,35]. The existing trichome ChIP-seq dataset provides four histone modification maps but no DNA methylation data at single-base resolution, leaving the relationship between TE-flanking CHH methylation and H3K27me3-to-H3K4me3 switching at CBDAS, THCAS, and PT4/CBGAS loci unresolved [27]. Second, the histone modification landscape across trichome development remains incomplete. The existing dataset covers H3K4me3, H3K56ac, H3K27me3, and H2A.Z in mature trichome tissue only [27]; a developmental series additionally integrating H3K36me3, H3K9me2, H3K27ac, and H3K9ac from trichome progenitor cells through secretory stage maturation would identify the full complement of chromatin-state transitions required to define rational epigenome editing targets. Third, stress-responsive epigenomic data in *C. sativa* are entirely absent. How drought, heat, UV-B, and light intensity reprogram the methylome and histone landscape at cannabinoid pathway gene loci is unknown. The only available stress-responsive epigenetic data in the *Cannabis* genus comes from UV-irradiated *C. indica* cell suspension cultures, which show genome-wide MSAP-detected hypermethylation coincident with approximately 4-fold CBDAS and THCAS upregulation but cannot resolve locus-specific methylation changes [55]. Promoter characterization confirms that *CsCBDAS* and *CsPT1* loci respond to IAA, GA3, SA, ABA, and abiotic stresses including heat, cold, light, salt, and drought, establishing that upstream signaling inputs converge on cannabinoid gene promoters; whether these signals act through chromatin-level mechanisms remains unresolved [59]. Fourth, transgenerational epigenetic inheritance in *Cannabis* has not been tested. Whether propagation-induced or environmentally induced methylation changes are reset between sexual generations or transmitted to progeny with consequences for cannabinoid profiles is unknown, and cultivar-specific differential methylated positions that accumulate during micropropagation have demonstrated potential phenotypic impact without confirmed heritability [28]. Fifth, the trichome chromatin-reading transcription factors that establish cannabinoid pathway gene locus identity remain unidentified. AP2/ERF, WRKY, bHLH, bZIP, MYB, and NAC family members modulate cannabinoid pathway expression through hormonal and elicitor signaling, but whether any of these interact specifically with H3K4me3-marked nucleosomes at CBDAS and THCAS promoters, or recruit Trithorax methyltransferases to these loci during trichome differentiation, has not been determined [60,61]. Each of these five gaps maps directly to a specific dCas9-effector targeting decision, covering methyltransferase, demethylase, HAT, HDAC, or chromatin remodeler deployment, and their experimental resolution defines the minimum knowledge base for rational *Cannabis* epigenome editing (Figure 2) [53,61].

## 4. CRISPR/dCas9-Based Epigenome Editing: Tools, Targets, and Implementation in *Cannabis*

**Figure 3:**
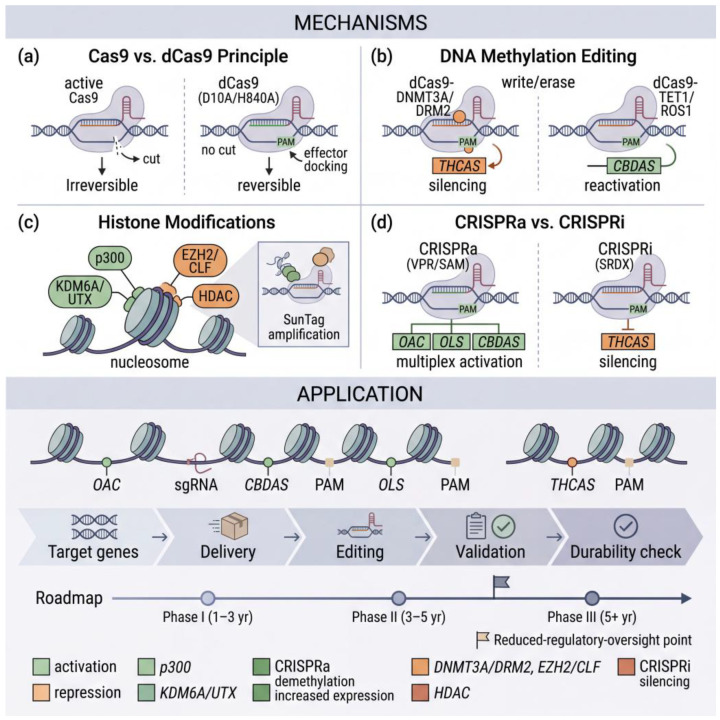
CRISPR/dCas9-based epigenome editing provides a reversible toolkit for engineering cannabinoid biosynthesis in *Cannabis sativa*. (**a**) Comparison of active Cas9 and catalytically dead Cas9 (dCas9). Active Cas9 cleaves target DNA to produce irreversible edits, whereas dCas9 (carrying the D10A and H840A substitutions) retains single-guide RNA (sgRNA)-directed, protospacer adjacent motif (PAM)-dependent targeting without cleavage and serves as a docking platform for effector domains, enabling reversible editing. (**b**) DNA methylation editing. dCas9 fused to DNMT3A or DRM2 writes methylation to silence *THCAS*, whereas dCas9 fused to TET1 or ROS1 erases methylation to reactivate *CBDAS*. (**c**) Histone modification editing. Effector domains tethered to dCas9 modify the nucleosome, with p300 and KDM6A/UTX promoting activation and EZH2/CLF and HDAC promoting repression; the SunTag array (inset) amplifies effector recruitment at a single target site. (**d**) Transcriptional modulation. dCas9-VPR/SAM mediates multiplex activation of *OAC*, *OLS*, and *CBDAS*, whereas dCas9-SRDX mediates silencing of *THCAS*. Next, application of the toolkit to cannabinoid biosynthesis genes. The target locus indicates sgRNA- and PAM-defined editing sites at *OAC*, *CBDAS*, *OLS*, and *THCAS* (**top**); the proposed experimental workflow proceeds from target gene selection through delivery, editing, validation, and durability assessment (**middle**); and the strategic roadmap spans Phase I (1 to 3 years), Phase II (3 to 5 years), and Phase III (5 or more years), with the flag denoting the point of potentially reduced regulatory oversight (**bottom**). Throughout, green and orange denote activating and repressive effectors or outcomes, respectively.

No epigenome editing experiment has been conducted in *Cannabis*. The trichome ChIP-seq atlas, the micropropagation methylation data, and the UV-B induction findings reviewed in Section 3 (Figure 2) collectively define at least six specific chromatin targets whose manipulation could alter cannabinoid pathway gene expression. The dCas9-effector toolkit has been validated in tomato, *Arabidopsis*, and *Nicotiana benthamiana* at secondary metabolite pathway loci with quantitative outcomes, and its application to *Cannabis* is technically feasible given existing transformation protocols (Figure 3; Table 2) [62]. What is required is a systematic mapping of which effector architectures are most appropriate for each *Cannabis* target, followed by a phased experimental program beginning in hairy root cultures and progressing to stable trichome-targeted transformation.

### 4.1. dCas9-Effector Architecture Determines Editing Specificity and Mark Durability

A catalytically dead Cas9 (dCas9), carrying D10A and H840A substitutions that inactivate both RuvC and HNH nuclease domains, retains full sgRNA-directed genomic targeting but cannot cleave DNA, providing a programmable sequence-specific anchor for epigenetic effector recruitment (Figure 3) [63]. The sgRNA base-pairs with a 20-nucleotide protospacer upstream of a 5′-NGG-3′ PAM, and multiple sgRNAs can be deployed simultaneously to coordinate epigenetic modification across all four cannabinoid pathway genes in a single transformation event [64]. The field of plant dCas9-based chromatin regulation has advanced substantially since these foundational reports, with tools now available for targeted deposition and removal of DNA methylation, H3K4me3, H3K9me2, H3K27me3, and H3K27ac at endogenous plant loci [65,66]. The SunTag recruitment system extends single dCas9 binding events into cooperative effector deposition by recruiting up to 24 copies of a single-chain antibody-effector fusion through a repetitive GCN4 peptide array, achieving histone mark deposition levels unattainable by single dCas9-effector fusions at loci with low chromatin accessibility (Figure 3) [67]. In *Arabidopsis*, SunTag-dCas9-VP64 activated endogenous targets more than 100-fold compared to dCas9-VP64 alone, and SunTag-dCas9-JMJ13 achieved targeted H3K27me3 removal at the CUC3 locus with developmental consequences, establishing that the SunTag architecture is the most effective amplification platform for both mark deposition and erasure in plants [30,68]. A modular dCas9-based recruitment platform enabling combinatorial epigenome editing has further demonstrated that coordinated deposition of multiple marks at a single locus produces synergistic transcriptional outcomes unachievable by single-effector systems [69]. The reversibility of epigenetically modified states, unlike permanent DNA sequence changes, means that editing outcomes can decay after dCas9-effector expression ceases unless mark maintenance machinery reinforces them, a property that is both a design challenge for durability and an opportunity for temporal control of pathway output [70].

### 4.2. DNA Methylation Editing at Cannabinoid Pathway Gene Promoters

Targeted CG methylation writing using dCas9 fused to the DNMT3A-DNMT3L catalytic domain silences target gene promoters by recruiting methyl-CpG-binding domain proteins and occluding transcription factor binding sites; in *Arabidopsis*, SunTag-dCas9-DNMT3A targeting endogenous loci achieved robust on-target CG methylation deposition with minimal detectable off-target methylation by whole-genome bisulfite sequencing [30]. Applied to *Cannabis*, dCas9-DNMT3A targeting the THCAS promoter in chemotype II cultivars could suppress THC branch competition and redirect CBGA flux toward CBDA production without altering the THCAS coding sequence, preserving the genetic integrity of the cultivar (Figure 3). Targeted CG demethylation using dCas9 fused to the TET1 catalytic domain oxidizes 5-methylcytosine stepwise to 5-hydroxymethylcytosine, 5-formylcytosine, and 5-carboxylcytosine, resolved to unmethylated cytosine through base excision repair; in *Arabidopsis*, this SunTag-dCas9-TET1cd system achieved heritable reactivation of the silenced FWA locus and targeted demethylation of the CACTA1 transposable element with minimal effects on global methylation patterns (Figure 3) [71]. The progressive CHG and CHH hypomethylation documented during *Cannabis* micropropagation represents a near-term application for dCas9-DNMT3A: targeted re-methylation of differentially methylated positions at cannabinoid pathway gene-associated loci in advanced-passage cultures could stabilize the epigenotype and potentially recover propagation-induced reductions in cannabinoid yield, a hypothesis testable in existing micropropagation systems by transient protoplast delivery of dCas9-DNMT3A ribonucleoprotein complexes [28].

### 4.3. Histone Modification Editing at Trichome Pathway Gene Loci

The H3K27me3 marks enriched at CBDAS, THCAS, OLS, and OAC loci in vegetative *Cannabis* tissues are an experimentally documented chromatin feature and therefore a candidate target for de-repression by dCas9 fused to the KDM6A or KDM6B JmjC demethylase domain, which actively removes H3K27me3 and restores transcriptional competency at Polycomb-silenced loci. In *Arabidopsis*, dCas9-JMJ13 SunTag-mediated targeting of the CUC3 boundary gene locus achieved H3K27me3 reduction with measurable developmental consequences (Figure 3), establishing that single-locus H3K27me3 erasure is sufficient to override Polycomb repression at a developmentally regulated plant gene [72]. Conversely, dCas9 fused to the p300 HAT catalytic domain deposits H3K27ac at target promoters; in *Arabidopsis*, dCas9-p300 targeted to the AREB1 promoter increased H3K27ac enrichment and activated drought-responsive gene expression, demonstrating that H3K27 acetylation written by dCas9-HAT is a viable transcriptional activation strategy at endogenous plant loci [73]. In *Cannabis*, dCas9-p300 targeted to the CBDAS or OAC promoters in trichome progenitor cells could amplify cannabinoid pathway flux by converting H3K27me3-marked bivalent states to H3K27ac-marked active states during the developmental window of trichome differentiation. The CMT3-H3K9me2 self-reinforcing loop at heterochromatic loci means that for *Cannabis* pathway genes flanked by transposable element-derived H3K9me2 regions, dual targeting of both the DNA methylation and histone modification layers simultaneously may be required for durable state changes; systematic profiling of available dCas9-based epigenome editors confirms that coordinated multi-layer editing produces more stable transcriptional outcomes than single-effector deployment [70].

### 4.4. Multiplexed CRISPRa and CRISPRi for Coordinated Pathway Modulation

The dCas9-VPR tripartite activation domain, combining VP64, p65, and Rta transactivation domains, has activated endogenous secondary metabolite pathway genes at endogenous loci in *Nicotiana benthamiana* and tomato; multiplexed CRISPRa activation of three flavonoid pathway genes simultaneously produced specific and predictable metabolite profiles without detectable off-target transcriptional changes by RNA-seq, establishing that CRISPRa is transferable across Solanaceae and potentially across broader crop phylogenies [30,74]. A multiplexed CRISPRa strategy using sgRNAs simultaneously targeting the CBDAS, OAC, and OLS promoters could amplify transcriptional flux through the CBD branch of the cannabinoid pathway across all three rate-limiting steps in a single transformation event, an approach that has no equivalent in conventional overexpression strategies because it avoids the dosage and codon optimization problems of transgene-based pathway engineering (Figure 3). CRISPRi using dCas9 fused to three tandem repeats of the SRDX EAR-motif repressor domain has silenced endogenous *Arabidopsis* genes with high efficiency and represents the most straightforward approach to suppressing THCAS expression in CBD-producing chemotype III cultivars without introducing a THCAS knockout mutation [64]. Combined deployment of CRISPRa at CBDAS and CRISPRi at THCAS in a single chemotype II cultivar provides a quantitative test of whether the CBD:THC chemotype ratio is primarily determined by the relative transcriptional outputs of these two competing synthases, a mechanistic question that has not been resolved experimentally (Table 2).

### 4.5. Delivery, Durability, and Validation Challenges Specific to Cannabis

Agrobacterium-mediated stable transformation of *Cannabis sativa* remains genotype-dependent and highly variable in efficiency across commercially relevant cultivars, representing the primary bottleneck for constitutive dCas9-effector expression [56,75]. The large size of dCas9-effector fusions, typically 160 to 240 kDa for SunTag architectures, imposes constraints on T-DNA cassette design and transformation efficiency relative to standard Cas9 constructs, and trichome-targeted expression requires a trichome-specific promoter whose regulatory elements have not been fully characterized in *Cannabis* [27,59]. Transgene-free ribonucleoprotein delivery into protoplasts avoids these constraints and is the recommended first-line delivery method for Phase I proof-of-concept experiments, enabling transient epigenome editing validation before stable transformation is attempted. Mark durability across vegetative propagation cycles is the most significant unresolved technical challenge: epigenetic marks introduced by dCas9-effectors must persist across the subculture generations used in commercial *Cannabis* propagation, yet the endogenous CMT3 and MET1 maintenance machineries may dilute or reset them during each cell division [70]. Dual-effector systems targeting both DNA methylation and histone marks at the same locus, or inducible dCas9-effector expression systems reactivated at defined intervals during propagation, represent the two most tractable strategies for maintaining editing outcomes across extended vegetative cycles. Validation of epigenome editing outcomes requires four measurements at each target locus: targeted bisulfite sequencing or CUT&RUN to confirm mark deposition or erasure, RT-qPCR to verify transcript-level response, targeted metabolite profiling by HPLC or LC-MS/MS to confirm cannabinoid phenotype, and whole-genome methylation and histone profiling to quantify off-target effects (Figure 3).

### 4.6. A Phased Experimental Program for Cannabis Epigenome Editing

The first priority is functional validation of dCas9-effector constructs in *Cannabis* using hairy root cultures and transient protoplast transfection, which circumvent stable transformation recalcitrance and allow rapid iteration of sgRNA design and effector selection. Specific first-phase targets are dCas9-TET1 demethylation of *CBDAS* promoter CG sites verified by targeted bisulfite sequencing and RT-qPCR, CRISPRa targeting of *OAC* and *OLS* to test transcriptional amplification of upstream polyketide flux, and CRISPRi targeting of *THCAS* in chemotype II hairy root cultures to quantify competitive pathway suppression. Once functional constructs are validated in hairy root systems, the second priority is stable transformation of two chemotype backgrounds using trichome-specific promoters derived from *THCAS* or *CsLTP1* regulatory sequences to drive trichome-targeted dCas9-effector expression, with methylation mark durability assessed by EM-seq at targeted loci across ten successive vegetative propagation cycles (Figure 3). The third priority addresses translational deployment: selection of stable epigenotype lines with verified cannabinoid profiles across developmental stages, assessment of epigenetic stability across sexual generations if breeding pipelines are used, and regulatory pathway evaluation in jurisdictions where non-mutagenic epigenome editing may qualify for reduced oversight relative to sequence-modifying technologies. Each phase generates specific quantitative outputs, methylation percentages, transcript fold-changes, cannabinoid yields, off-target profiling data, that define the go/no-go criteria for progression to the next phase.

**Table 2:** Proposed dCas9-based epigenome-editing strategies for the *Cannabis* cannabinoid pathway. The listed effects are working hypotheses; no epigenome-editing experiment has yet been performed in *Cannabis*.

Effector (dCas9 Fusion)	Molecular Action	Model-System Precedent	Proposed *Cannabis* Target	Hypothesized Effect (Untested in *Cannabis*)
dCas9-DNMT3A/3L (SunTag)	Writes CG methylation; silences promoters	On-target CG methylation at *Arabidopsis* loci [30]	*THCAS* promoter (chemotype II)	Suppress THC branch; redirect CBGA flux to CBDA
dCas9-TET1 (SunTag)	Removes CG methylation (oxidation + BER)	Heritable reactivation of *FWA*; demethylation of *CACTA1* in *Arabidopsis* [56]	Methylated pathway-gene promoters	Reactivate silenced biosynthetic loci
dCas9-p300	Writes H3K27ac; activates transcription	dCas9-p300 at *AREB1* activated drought genes in *Arabidopsis* [30]	*CBDAS* or *OAC* promoters (trichome progenitors)	Amplify cannabinoid pathway flux
dCas9-KDM6A/KDM6B (JMJ13)	Removes H3K27me3; relieves Polycomb repression	dCas9-JMJ13 reduced H3K27me3 at *CUC3* in *Arabidopsis* [72]	H3K27me3-marked *CBDAS*, *THCAS*, *OLS*, *OAC*	De-repress Polycomb-silenced pathway genes
CRISPRa (dCas9-VPR/SunTag-VP64)	Recruits transactivators; upregulates genes	Multiplexed activation of flavonoid genes in *Nicotiana*/tomato [74]	*CBDAS*, *OAC*, *OLS* promoters (multiplexed)	Amplify flux through CBD branch at three steps
CRISPRi (dCas9-3xSRDX)	EAR-motif repression; silences genes	High-efficiency silencing in *Arabidopsis* [64]	*THCAS* (chemotype III)	Suppress *THCAS* without a knockout mutation

## 5. Conclusions

Three bodies of evidence now anchor *Cannabis* epigenomics as a field with direct implications for cannabinoid yield engineering. H3K4me3 and H3K56ac co-occupy the promoters and gene bodies of cannabinoid biosynthetic genes exclusively in glandular trichome tissue, while H3K27me3 marks the same loci in leaf and stem, establishing that trichome-specific cannabinoid gene expression is controlled by a Polycomb-to-Trithorax chromatin switch rather than by transcription factor availability alone. Progressive CG and CHG differential methylation accumulate during micropropagation in a cultivar-specific pattern, with promoter-region changes reaching up to 22% by 20 subcultures, identifying DNA methylation maintenance as a candidate determinant of epigenetic stability in clonally propagated *Cannabis* material; whether this epigenetic drift alters cannabinoid output has not yet been tested against metabolite data. UV irradiation of *Cannabis indica* cell suspension cultures increases genome-wide DNA methylation and elevates CBDAS and THCAS transcript levels approximately 4-fold (Figure 2), demonstrating that environmentally triggered methylation changes are coupled to cannabinoid pathway gene expression in the *Cannabis* genus. These three findings define six specific chromatin targets for dCas9-effector intervention: the H3K27me3 repressive state at cannabinoid loci in non-trichome tissues, addressable by dCas9-JMJ13 or dCas9-KDM6A; the H3K27me3-to-H3K4me3 transition in trichome progenitors, addressable by dCas9-p300; the CG hypomethylation accumulating at pathway gene-associated loci during micropropagation, addressable by dCas9-DNMT3A; the competitive THCAS transcriptional activity in CBD-producing chemotypes, suppressible by dCas9-3xSRDX CRISPRi; the upstream OAC and OLS transcriptional bottleneck, amplifiable by dCas9-VPR or dCas9-SunTag CRISPRa; and the stress-responsive methylation-cannabinoid induction axis, exploitable through dCas9-DRM2 targeting at UV-responsive loci (Table 2). Each target is supported by experimental evidence from at least one plant model system; none has been tested in *Cannabis*. Five knowledge gaps must be addressed before these strategies can be rationally deployed. No comprehensive, whole-genome base-resolution methylome exists for glandular trichomes or other cannabinoid-relevant *Cannabis* tissues; the existing base-resolution data come from reduced-representation enzymatic methyl sequencing of micropropagated shoots and do not provide whole-genome coverage of the relevant cell types. The trichome histone modification atlas covers only four marks in mature tissue and lacks a developmental series from progenitor to secretory stage. No stress-responsive epigenomic dataset exists for *C. sativa* at locus resolution. The transcription factors reading H3K4me3 at cannabinoid pathway gene promoters in trichomes remain unidentified. Whether propagation-induced methylation changes persist across sexual generations or are reset in progeny has not been determined. Each gap maps directly to a tractable experimental design: EM-seq in trichome-enriched tissue, developmental CUT&RUN from progenitor to mature secretory trichome, locus-resolved bisulfite sequencing under abiotic stress, chromatin immunoprecipitation with MYB, AP2/ERF, and WRKY antibodies at cannabinoid pathway gene promoters, and methylation profiling across F1 progeny of hypomethylated propagation lines. *C. sativa* epigenome editing does not require resolution of all five gaps before the first experiment is attempted. Functional validation of dCas9-SunTag-JMJ13 at the CBDAS locus in hairy root cultures, confirmed by CUT&RUN and RT-qPCR, is achievable with existing tools and existing *Cannabis* transformation protocols. That single experiment would establish whether H3K27me3 erasure at a cannabinoid pathway gene promoter is sufficient to elevate transcript levels in a non-trichome tissue context, providing the first direct causal evidence linking a specific chromatin mark to cannabinoid pathway gene expression in *Cannabis*. This is the experiment the field needs next.

## Figures and Tables

**Table 1:** Reported epigenetic regulation of secondary-metabolite and cannabinoid biosynthesis in plants.

Epigenetic Mechanism	System	Target Genes/Locus	Key Finding	Reference
DNA methylation (CHH)	*Papaver somniferum*	Alkaloid biosynthetic loci	Cultivar-specific differences in alkaloid yield correlate with differential CHH methylation	[23,24]
Histone acetylation	*Artemisia annua*	Artemisinin pathway	Jasmonate-induced histone acetylation implicated in pathway activation	[25,26]
TF/chromatin control	*Catharanthus roseus*	*STR*, *TDC* promoters	Jasmonate-responsive bHLH factors activate alkaloid promoters via JAZ derepression	[20]
Histone modification (H3K4me3, H3K56ac vs. H3K27me3)	*Cannabis sativa* (trichome vs. leaf/stem)	*CBDAS*, *PT4*/*CBGAS*, terpenoid synthases	Activating marks occupy pathway promoters in trichomes but are absent in leaf/stem; reciprocal H3K27me3 in vegetative tissue	[27]
DNA methylation (CG, CHG)	*Cannabis sativa* (micropropagation)	Promoter and intergenic regions	Cultivar-specific accumulation of differentially methylated positions over 20 subcultures; yield effect not yet established	[28]
DNA methylation (genome-wide, MSAP)	*Cannabis indica* (cell suspension, UV)	Genome-wide; locus not resolved	UV increases genome-wide methylation and elevates *CBDAS*/*THCAS* transcripts; promoter-specific methylation not demonstrated	[55]

## Data Availability

No new data were created or analyzed in this study. Data sharing is not applicable to this article.

## Abbreviations

3D-GBS: three-dimensional genotyping by sequencing; AaHAT: *Artemisia annua* histone acetyltransferase; ABA: abscisic acid; ADS: amorpha-4,11-diene synthase; AP2/ERF: APETALA2/Ethylene Response Factor; ATAC-seq: assay for transposase-accessible chromatin with sequencing; bHLH: basic helix-loop-helix; BRM: Brahma; bZIP: basic leucine zipper; Cas9: CRISPR-associated protein 9; CBDA: cannabidiolic acid; CBDAS: cannabidiolic acid synthase; CBGA: cannabigerolic acid; CBD: cannabidiol; CG: cytosine–guanine methylation context; CHD: chromodomain helicase DNA-binding; CHG: cytosine–(any base except G)–guanine methylation context; CHH: cytosine–(any base except G)–(any base except G) methylation context; ChIP-seq: chromatin immunoprecipitation sequencing; CLF: Curly Leaf; CMT3: Chromomethylase 3; CRISPR: clustered regularly interspaced short palindromic repeats; CRISPRa: CRISPR activation; CRISPRi: CRISPR interference; CRF35: Chromatin Remodeling Factor 35; CsHDA1/5/7: *Cannabis sativa* Histone Deacetylase 1/5/7; CsSRT1: *Cannabis sativa* Sirtuin 1; CYP71AV1: cytochrome P450 71AV1; CYP80B1: cytochrome P450 80B1; dCas9: catalytically dead Cas9; DBR2: double bond reductase 2; DME: Demeter; DMPs: differentially methylated positions; DNMT3A: DNA methyltransferase 3A; DRM2: Domains Rearranged Methyltransferase 2; ELF6: Early Flowering 6; EM-seq: enzymatic methyl sequencing; EZH2: Enhancer of Zeste Homolog 2; GA3: gibberellin A3; GCN5/HAG1: General Control Non-repressible 5/Histone Acetyltransferase of the GNAT family 1; GOT: geranylpyrophosphate:olivetolate geranyltransferase; GPP: geranyl pyrophosphate; H2A.Z: histone H2A variant Z; H3K4me3: histone H3 lysine 4 trimethylation; H3K9ac: histone H3 lysine 9 acetylation; H3K9me2: histone H3 lysine 9 dimethylation; H3K27ac: histone H3 lysine 27 acetylation; H3K27me3: histone H3 lysine 27 trimethylation; H3K36me3: histone H3 lysine 36 trimethylation; H3K56ac: histone H3 lysine 56 acetylation; HAT: histone acetyltransferase; HDA6: Histone Deacetylase 6; HDA19: Histone Deacetylase 19; HDAC: histone deacetylase; HPLC: high-performance liquid chromatography; IAA: indole-3-acetic acid; JAZ: Jasmonate ZIM-domain repressor; KDM6A/UTX: Lysine Demethylase 6A; KYP/SUVH4: Kryptonite/Suppressor of Variegation Homolog 4; LC-MS/MS: liquid chromatography–tandem mass spectrometry; LEC1/LEC2: Leafy Cotyledon 1/2; MEA: Medea; MeJA: methyl jasmonate; MEP: methylerythritol phosphate; MET1: DNA Methyltransferase 1; MIA: monoterpene indole alkaloid; MSAP: methylation-sensitive amplified polymorphism; MYB: Myeloblastosis transcription factor; NAC: NAM/ATAF/CUC domain transcription factor; nt: nucleotide; OA: olivetolic acid; OAC: olivetolic acid cyclase; OLS: olivetol synthase; ORCA: octadecanoid-responsive *Catharanthus* AP2-domain transcription factor; p300: histone acetyltransferase p300; PAM: protospacer adjacent motif; PKL: Pickle; PRC2: Polycomb Repressive Complex 2; PT4/CBGAS: prenyltransferase 4/cannabigerolic acid synthase; RdDM: RNA-directed DNA methylation; REF6: Relative of Early Flowering 6; RNA-seq: RNA sequencing; ROS1: Repressor of Silencing 1; RT-qPCR: reverse-transcription quantitative polymerase chain reaction; SA: salicylic acid; SAM: Synergistic Activation Mediator; SDG8/ASHH2: SET Domain Group 8/Ash1 Homolog 2; sgRNA: single guide RNA; siRNA: small interfering RNA; SRDX: Super-repressor Domain X; STR: strictosidine synthase; SunTag: SuperNova Tagging system; SWI/SNF: SWItch/Sucrose Non-Fermentable; SWN: Swinger; T-DNA: transfer DNA; TDC: tryptophan decarboxylase; TE: transposable element; TET1: Ten-eleven translocation methylcytosine dioxygenase 1; THCA: Δ9-tetrahydrocannabinolic acid; THCAS: tetrahydrocannabinolic acid synthase; THC: Δ9-tetrahydrocannabinol; TKS: tetraketide synthase; TrxG: Trithorax group complex; TSA: trichostatin A; T6ODM: thebaine 6-O-demethylase; VPR: VP64-p65-Rta; WRKY: WRKY domain transcription factor.
